# Perinatal Exposure to Methoxychlor Affects Reproductive Function and Sexual Behavior in Mice

**DOI:** 10.3389/fendo.2020.00639

**Published:** 2020-09-09

**Authors:** Mariangela Martini, Pascal Froment, Isabelle Franceschini, Delphine Pillon, Edith Guibert, Claude Cahier, Sakina Mhaouty-Kodja, Matthieu Keller

**Affiliations:** ^1^Physiologie de la Reproduction et des Comportements, UMR 7247 INRA/CNRS/Université François Rabelais, Nouzilly, France; ^2^Department of Biological Sciences & Toxicology Program, North Carolina State University, Raleigh, NC, United States; ^3^Unité Expérimentale de Physiologie Animale de l'Orfrasière, UE 1297, INRA, Nouzilly, France; ^4^Sorbonne Université, CNRS, INSERM, Neuroscience Paris Seine - Institut de Biologie Paris Seine, Paris, France

**Keywords:** endocrine disruptor, reproduction, hormones, sexual behavior, kisspeptin

## Abstract

Numerous chemicals derived from human activity are now disseminated in the environment where their exert estrogenic endocrine disrupting effects, and therefore represent major health concerns. The present study explored whether Methoxychlor (MXC), an insecticide with xenoestrogens activities, given during the perinatal period (from gestational day 11 to postnatal day 8) and at an environmentally dose [20 μg/kg (body weight)/day], would affect reproductive physiology and sexual behavior of the offspring in mice. While MXC exposure did not induce any differences in the weight gain of animals from birth to 4 months of age, a clear difference (although in opposite direction according to the sexes) was observed on the anogenital distance between intact and exposed animals. A similar effect was also observed on preputial separation and vaginal opening, which reflects, respectively, in males and females, puberty occurrence. The advanced puberty observed in females was associated with an enhanced expression of kisspeptin cells in the anteroventral periventricular region of the medial preoptic area. Exposure to MXC did not induce in adult females changes in the estrous cycle or in the weight of the female reproductive tract. By contrast, males showed reduced weight of the epididymis and seminiferous vesicles associated with reduced testosterone levels and seminiferous tubule diameter. We also showed that both males and females showed deficits in mate preference tests. As a whole, our results show that MXC impacts reproductive outcomes.

## Introduction

Methoxychlor (MXC) is an insecticide that is acting as an Endocrine Disrupting Compound (EDC). MXC is persistent in the environment (1) because significant amounts of MXC and deriving metabolites can be measured in human tissues, long after stopping its use (2). For example, MXC has been found in human tissue samples (3) and breast milk (4). It has been demonstrated recently that MXC has the potential to promote the transgenerational inheritance of disease (5). Thus, the health effects of MXC remain an important public health concern in humans.

MXC is known to act as an agonist on estrogen receptor (ER) α and ERβ and as an antagonist of androgen receptor [AR (6)]. MXC contamination seems to occur mainly through ingestion: MXC is absorbed at the level of the gastrointestinal tract and is then metabolized in the liver, following a detoxification pathway (6). Even though MXC has a relatively low binding affinities for ERα and ERβ (RBAs < 0.01), it is metabolized in the liver and its estrogenic activity is carried out by its various metabolites. Its major metabolite is 2,2-bis(p-hydroxyphenyl)-1,1,1-trichloroethane (HPTE) which is even higher effective ERs agonists than MXC itself (7, 8).

The neonatal period is a period of high sensitivity to endocrine disruption. Indeed, detrimental factors can impact the developmental trajectory of organisms and as a consequence, compromise their adult health. In this context, it has been shown that MXC exposure affects reproductive function in mice: neonatal treatments cause abnormal morphology of ovarian and reproductive tract in adult females (9, 10). In males, neonatal MXC treatment inhibits the development of accessory reproductive organs and plasma testosterone levels (11, 12). Similar effects have been also documented in rats (6, 13–15).

In rodents, brain sexual differentiation occurs during the late gestational and early neonatal periods. This process is finely tuned by sex hormones and is highly sensitive to exposure to EDCs with androgenic and/or estrogenic properties. In males, testosterone from fetal and neonatal testes permanently potentiates male and inhibits female behavioral and neuroanatomical characteristics (16). Such organizational effects of testosterone induces the expression of male-typical behaviors in adulthood (such as preference for a receptive female, mounts and thrusts) and conversely the inability to adopt the female receptive posture (lordosis). Testosterone acts also perinatally to suppress the neuroanatomical characteristics required for the induction of the ovulatory surge of luteinizing hormone (LH) (17, 18). These effects are mainly induced by estradiol after the neural aromatization of testosterone.

In this context, it has been shown that perinatal MXC exposure can interfere with behavioral and sexual differentiation (19–23). However, it should be noticed that most if not all of the previous studies on the effects of perinatal exposure to MXC on reproductive function or behavior were performed at very high doses [usually within the range 100 μg/kg/day to several mg/kg/day, for example: (10, 24, 25)] and on rather short exposure durations [for example 3 days: (10, 25)]. It is clear that these conditions do not reflect the natural exposure to EDCs that usually act at very low environmental doses and during quite chronic durations of exposure.

Therefore, we decided here to investigate the consequences of perinatal exposure to a low dose of MXC (20 μg/kg/day, well below the doses used previously to study the effects of MXC on reproductive function) and during a more long-term exposure, because these conditions mimic more closely the natural exposition to this EDC during the perinatal period. We explored the consequences on reproductive physiology and sexual behavior (especially mate preference) in both male and female mice.

## Materials and Methods

### Maternal Treatment and Procedure

General treatments and housing of animals were performed as already described (26). Briefly, Swiss CD1 mice were purchased from Janvier Breeding Center (Le Genest Saint-Isle, France) and bred in our animal facility. Adult (2–3 months old) virgin females were time-mated by being placed into the cage of a stud male for one night, beginning at 18h00 (at the end of the light phase of the 12-h light/dark cycle). Mating was verified by the vaginal plug presence (gestational day 0). After mating, pregnant females were housed three per cage (45 × 25 × 15 cm^3^) until the treatment started.

MXC was administered at 20 μg/kg (body weight)/day, according to the dose chosen in previous experiments (19, 21, 22, 26). This dose is below the 50 mg/kg/day lowest observed adverse effect level (LOAEL) and the 5 mg/kg predicted no observed adverse effect level [NOAEL; (27)]. This dose is also within the human exposure range and is considered as not teratogenic (28–31). Pregnant dams were daily trained to drink a small volume (0.1 ml) of sesame oil from a micropipette to ensure that the treatment procedure was not generating stress. On gestational day 11, females were individually housed and randomly assigned to one of the following treatment groups: sesame oil (control; *N* = 15) or MXC at 20 μg/kg/day (Sigma Chemical; *N* = 15) dissolved in sesame oil. Each female was fed 0.1 ml/50 g body weight/day of sesame oil with or without MXC 4 h after light onset and from gestational day 11 to post-natal day (PD) 8. During this critical period for brain development in mice and rats, estrogens organize permanently and irreversibly specific brain circuitries (32, 33).

At birth, litters were sexed, culled to 10 pups (5 ± 1 males and 5 ± 1 females) and returned to their mothers within 12 h of postnatal life. The offspring were weaned on PD 21 and group-housed with same-sex littermates (5 mice/cage) at 22 ± 1°C, under a 12-h reversed dark/light cycle (lights off at 10h00). *Ad libitum* water and pellet food (Safe, Augy, France) were provided. The study was performed in accordance with the French and European legal requirements (Decree 2010/63/UE).

### Experimental Groups

Behavioral tests were performed between 10h00 and 18h00 on one cohort of male and female control (OIL) and treated (MXC) mice. Two different cohorts of animals were used for blood collection and necropsy and for kisspeptin immunofluorescence labeling, respectively. All the animals were evaluated for weight gain, reproductive development and pubertal indices. To avoid any litter effect, a maximum of two pups originating from the same litter were included in the same experimental group for the evaluation of weight gain, reproductive development and pubertal indices and a maximum of one pup originating from the same litter was included in the same experimental group for blood collection, necropsy, immunofluorescence labeling and behavioral experiments.

To avoid that female behavioral responses were influenced by their hormonal status (34, 35), the phase of the estrous cycle was determined by vaginal smear cytology for each female. Only females at diestrus stage, were used for necropsy and immunofluorescence labeling. Since we needed sexually receptive females for the mate preference tests, only females at estrus stage were used in this test.

### Evaluation of Weight Gain, Reproductive Development, and Puberty Onset

From postnatal day (PD) 4 to PD 33 the growth and the anogenital distance (AGD) of male (OIL *N* = 30; MXC *N* = 34) and female (OIL *N*=30; MXC *N*=35) mice were monitored by weighing animals every week and subsequently, once a month until PD 120. AGD was measured using a caliper with a digital readout.

Pubertal landmarks were measured as indices of maturation of the gonadotrope axis. Puberty onset was evaluated through daily visual examination in females (*N* = 30 each group) by assessing the age at vaginal opening and in males (*N* = 30 each group) by assessing the age at preputial separation (36). Measurement of these indices began on PD 21.

### Kisspeptin Immuofluorescence Labeling

At 2 months of age, a different cohort of animals (*N* = 5 each group) was used to detect kisspeptin immunofluorescence. Adult animals were anesthetized and perfused with 1% sodium nitrite in phosphate buffer saline, followed immediately by 4% cold paraformaldehyde solution in 0.1 M phosphate buffer (pH 7.4). Brains were quickly dissected and post-fixed in 4% paraformaldehyde for 2 h and were then placed overnight in a sucrose solution in PBS, frozen in liquid isopentane at −35°C and stored in a deep freezer at −80°C.

Brains were then serially cut in the coronal planes at 30 μm thickness with a cryostat. The plane of sectioning was oriented to match the drawings corresponding to the coronal sections of the mouse brain atlas (37). Serial sections were collected in a cryoprotectant solution at −20°C (38). Every fourth section (a section every 120 μm) was processed for kisspeptin immunofluorescence labeling (sheep anti-kisspeptin antibody AC053 [1:10 000]) as previously described (39–41). Kisspeptin cell numbers were analyzed by counting the number of kisspeptin cell bodies in the anteroventral periventricular nucleus (AVPV) using anatomically matched section identified using the Mouse Brain Atlas of Franklin and Paxinos [(37) plates 28–29; two sections per animal]. We did not quantify kisspeptin cell bodies in the arcuate nucleus, because the high density of fibers prevented the visualization of cell bodies and their quantification.

### Necropsy and Histology of Reproductive Organs

In adult female mice, we first measured the length of the ovarian cycle and of its various phases by vaginal smear cytology as previously described. Vaginal smears were performed daily during 3 weeks in both MXC and OIL females. To measure fertility, MXC (*n* = 6) and OIL (*n* = 6) females were paired over 3 months with sexually active males. The number of pups produced and their sex-ratio were recorded. Finally, we weighted uterus and ovaries as described in the following paragraph.

After blood collection, the animals were necropsied and internal abdominal organs examined. The following organs were dissected out and weighed: in females, ovaries (paired, OIL *N* = 8; MXC *N* = 9) and uterus (OIL *N* = 13; MXC *N* = 12); in males, testis (paired, *N* = 12 each group), epididymis (paired, *N* = 12 each group), coagulating glands (paired, *N* = 12) and seminiferous vesicles (paired, *N* = 12). One testis was immediately recovered and fixed in Bouin solution for histological studies: testes were processed in paraffin and serially sectioned at 7 μm slice thickness. Sections were stained with hematoxylin and eosin. The round or nearly round transverse section of seminiferous tubules diameters were measured for each testis using an ocular measuring device (*n* = 30 measurements per animal; with 5 control and 5 treated animals). Another set of sections were deparaffinized, then hydrated and permeabilizated with Proteinase K (FragEL kit, Calbiochem, VWR, West Chester, PA, USA). Sections were incubated in H_2_O_2_ 3% to inactivate endogenous peroxydases, then the *in situ* end labeling of nuclear DNA fragmentation was performed in a humid chamber for 1.5 h at 37°C by a terminal deoxynucleotidyltransferase (TdT). Staining was revealed with 3,3′-diaminobenzidine (DAB). Negative controls were free of terminal deoxynucleotidyltransferase. Sections were stained with hematoxylin and eosin before qualitative observation under light microscopy.

### Testosterone Assay

At scheduled necropsy, additional male mice (OIL *N* = 12; MXC *N* = 12) were weighed and blood was collected in males after decapitation under anesthesia (Ketamine/Dormitor) in a 9-ml serum separation tube and left at room temperature for 30 min. Serum was then collected by centrifuging the blood for 20 min at a speed of 3,000 rpm and a temperature of 4°C. Serum was subsequently stored at −70°C and later analyzed for testosterone content using a method adapted from the assay described previously (41, 42). Results were reported as ng/ml.

In addition, the testes that was not used for histology, was homogenized in cold Phosphate buffered saline (PBS) without Ca^2+^ and Mg^2+^ by using an ultrathurax as described by Keene et al. (43). The testicular extracts were used to measure the intratesticular testosterone concentrations by radioimmunoassay. Samples were assayed in duplicate as previously described (42). Sensitivity of testosterone assay was 15 pg/tube and intra-assay coefficients of variation 5.3%.

### Mate Preference Tests

To evaluate the role of MXC exposure on sexual behavior, we decided to explore sexual preferences. Tests were conducted in a Plexiglas Y-maze apparatus for 5 min [previously described in (44, 45)]. When animals were tested for mate recognition using volatile body odors as odor stimuli, removable Plexiglas doors were placed at the distal end of each arm to separate the goal boxes from the rest of the maze. Volatile body odors were derived from placing an intact anesthetized male and an estrous female behind these opaque doors. The time that the mouse spent investigating the stimulus in nasal contact with the opaque partitions was recorded.

Three days before the test, male (*N* = 11 in each group) and female (OIL *N* = 11; MXC *N* = 10) mice were accustomed to the Y-maze apparatus for 5 min in the absence of any odor stimulus. At the beginning of each test, the subject was placed in the start box with the door closed to adapt for 1 min. The test began when the door was removed and the subject could freely move around in the Y-maze. The maze was cleaned with 70% ethanol between trials. The same mice were also tested for their preference toward volatile odors emanating from two different soiled beddings, intact male- vs. estrous female-soiled beddings.

### Statistical Analysis

Effects of MXC in male and female mice were compared using analyses of variance (ANOVA), with sex (Male vs. Female) and perinatal exposure (Oil vs. MXC) and/or postnatal days as independent factors. *Post hoc* Fisher's LSD tests were used to further explore differences revealed by ANOVAs. Cumulative percentage of females showing vaginal opening and males showing preputial separation at each PD was compared between experimental groups by the χ^*2*^ test of Pearson. Time spent sniffing the two olfactory stimuli and the two stimulus animals during mate preference tests, respectively, and the weight of reproductive organs were compared using paired *t*-test within experimental groups.

All the analyses were performed using the software Statistica 6.0 (StatSoft Inc., Tulsa, OK, USA). Differences were considered significant when *p* < 0.05. Data are represented as mean ± SEM.

## Results

### Evaluation of Weight Gain, Puberty Onset, and Reproductive Development

**Figure 1:**
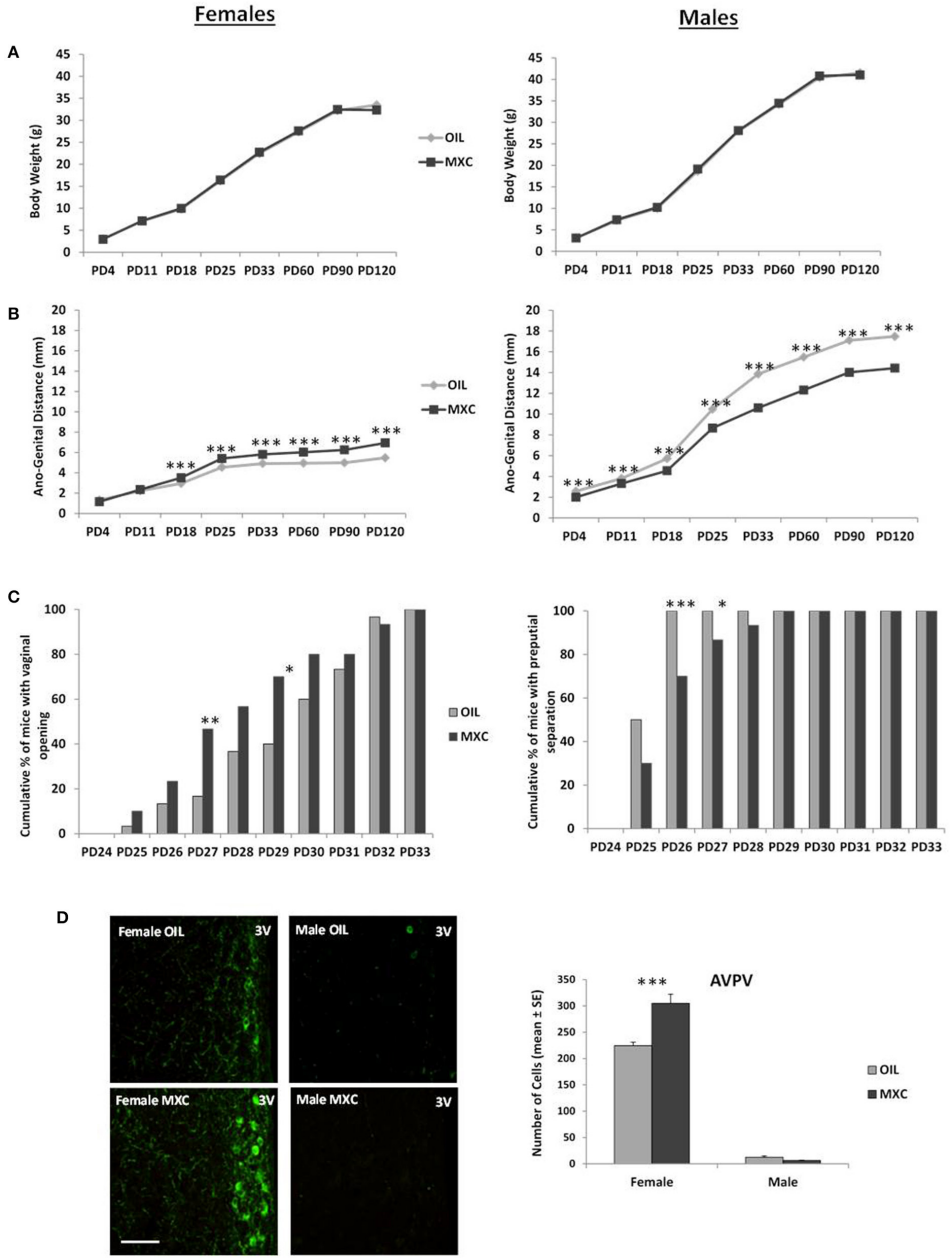
Effect of perinatal exposure to MXC developmental parameters in both male and female mice. **(A)** Body weight gain from PD4 to PD120 shows no difference according to perinatal exposure to MXC or oil; **(B)** by contrast, ano-genital distance (AGD) between PD4 and PD120 was affected by MXC exposure, with a larger AGD distance found in females from PD18 and a shorter AGD found in males from PD4; **(C)** pubertal transition measured through vaginal opening (VO) in female mice (cumulative percentage) and preputial separation in male mice (cumulative percentage) also showed an effect of MXC exposure: MXC exposed females showed a higher percentage of VO at PD27 and PD29 in comparison to control females, while males exposed to MXC showed a lower percentage of preputial separation at PD26 and PD27; **(D)** these results on pubertal transition were in accordance with adult immunocytochemical expression of Kisspeptin, a molecular switch of importance for pubertal transition in mice. For all graphs: *p < 0.05; **p < 0.01, ***p < 0.001 for OIL vs. MXC exposure.

Three-way repeated-measures ANOVA with sex and perinatal exposure as independent factors and PD as repeated factor revealed a significant effect of sex on body weight [*F*_(1,125)_ = 589.27, *p* < 0.001], since males had higher body weight than females (Figure 1A). There was no effect of treatment [*F*_(1,125)_ = 0.160, *p* = 0.690] or interaction between sex and treatment [*F*_(1,125)_ = 0.249, *p* = 0.619]. However, there was a significant effect of PD [*F*_(7,875)_ = 9450.6, *p* < 0.001] and a significant interaction between sex and PD [*F*_(7,875)_ = 175.60, *p* < 0.001] on body weight.

Regarding the AGD, three-way repeated measures ANOVA, with sex and treatment as independent factors and PD as repeated factor, showed a significant effect of sex, treatment and PD [sex: *F*_(1,125)_ = 15579, *p* < 0.001; treatment: *F*_(1,125)_ = 225.34, *p* < 0.001; PD: *F*_(7,875)_ = 9147.1, *p* < 0.001] and a significant interaction sex^*^treatment and sex^*^treatment^*^PD [sex^*^treatment: *F*_(1,125)_ = 1048.7, *p* < 0.001; sex^*^treatment^*^PD: *F*_(7,875)_ = 130.16, *p* < 0.001]. MXC had a significant effect both in males and in females on AGD from PD 4 to PD 120, with males having a longer AGD than females, treated males having a shorter AGD than control males and treated females having a longer AGD than controls (Figure 1B).

Regarding puberty onset, females perinatally exposed to MXC showed the same vaginal opening pattern than control females, distributed over 9 days (from PD 25 to PD 33; Figure 1C). However, the χ^2^ test revealed significant differences in the cumulative percentage of female mice with vaginal opening between the two experimental groups in PD 27 and PD 29 (PD 27: χ^*2*^_2_ = 6.2388, *p* = 0.0125; PD 29: χ^*2*^_2_ = 5.4545, *p* = 0.0195). The greatest difference between the two groups was observed at PD 27: the percentage of females showing vaginal opening was 3 times higher in the group perinatally exposed to MXC in comparison to the groups of control females. As a whole, females exposed to MXC had a slightly earlier vaginal opening pattern than control females.

Males perinatally exposed to MXC showed a longer preputial separation pattern, distributed over 5 days (from PD 25 to PD 29) whereas control males showed a shorter preputial separation pattern extending over 2 days (PD 25–PD 26; Figure 1C). The χ^2^ test revealed significant differences in the cumulative percentage of male mice with preputial separation between the two experimental groups in PD 26 and PD 27 (PD 26: χ^*2*^_2_ = 10.5882, *p* = 0.0011; PD 27: χ^*2*^_2_ = 4.2857, *p* = 0.0384). As a whole, males perinatally exposed to MXC had later preputial separation than control ones.

### Kisspeptin Immunofluorescence Labeling

A two-way ANOVA, with sex and perinatal treatment as independent factors, revealed a significant effect of sex [*F*_(1,20)_ = 754.20, *p* < 0.001], a significant effect of treatment [*F*_(1,20)_ = 15.898, *p* < 0.001] and a significant interaction sex*treatment [*F*_(1,20)_ = 21.792, *p* < 0.001]. Subsequent *post hoc* Fisher's LSD analysis revealed a significant greater number of Kisspeptin neurons in AVPV in females compared to males (*p* < 0.001). In addition, perinatal MXC treatment increased the number of Kisspeptin neurons in females (*p* < 0.001), but had no effects in males (*p* = 0.635) (Figure 1D).

### Female Reproductive Function

**Figure 2:**
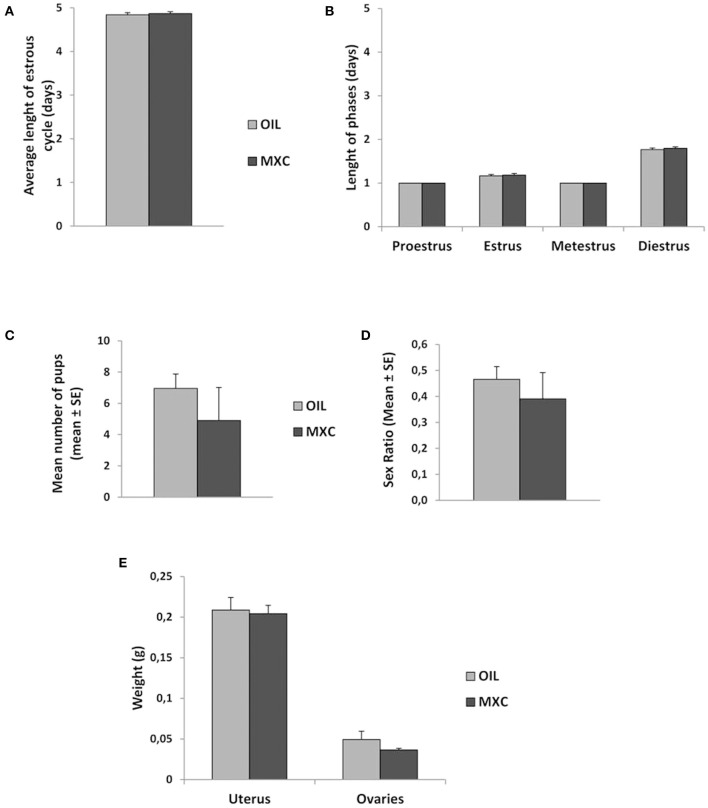
Effect of perinatal exposure to MXC on female reproductive parameters. MXC exposure did not induce any effect in comparison to control females in average length of estrous cycle [both general length **(A)** and length of each phase **(B)** of the estrous cycle]. When paired with a sexually active male over a 3-months period, MXC treated females did not produce a different number of pups than control females and the sex ratio of these pups did also not vary **(C,D)**. Finally, no difference between uterus and ovaries weight was observed between MXC and OIL treated females **(E)**.

Measures of length of ovarian cycle did not reveal any differences according to treatment (Figures 2A,B). When pairing males with females during 3 months, no differences in the mean number of pups produced neither in their sex ratio was observed (Figures 2C,D). *T*-test revealed also no effects of MXC exposure in uterus and ovaries weight between MXC exposed and control females (*t*-value: 0.230, df: 23, p = 0.820 and *t*-value: 1.288, df: 15, *p* = 0.217, respectively) (Figure 2E).

### Male Reproductive Function

**Figure 3:**
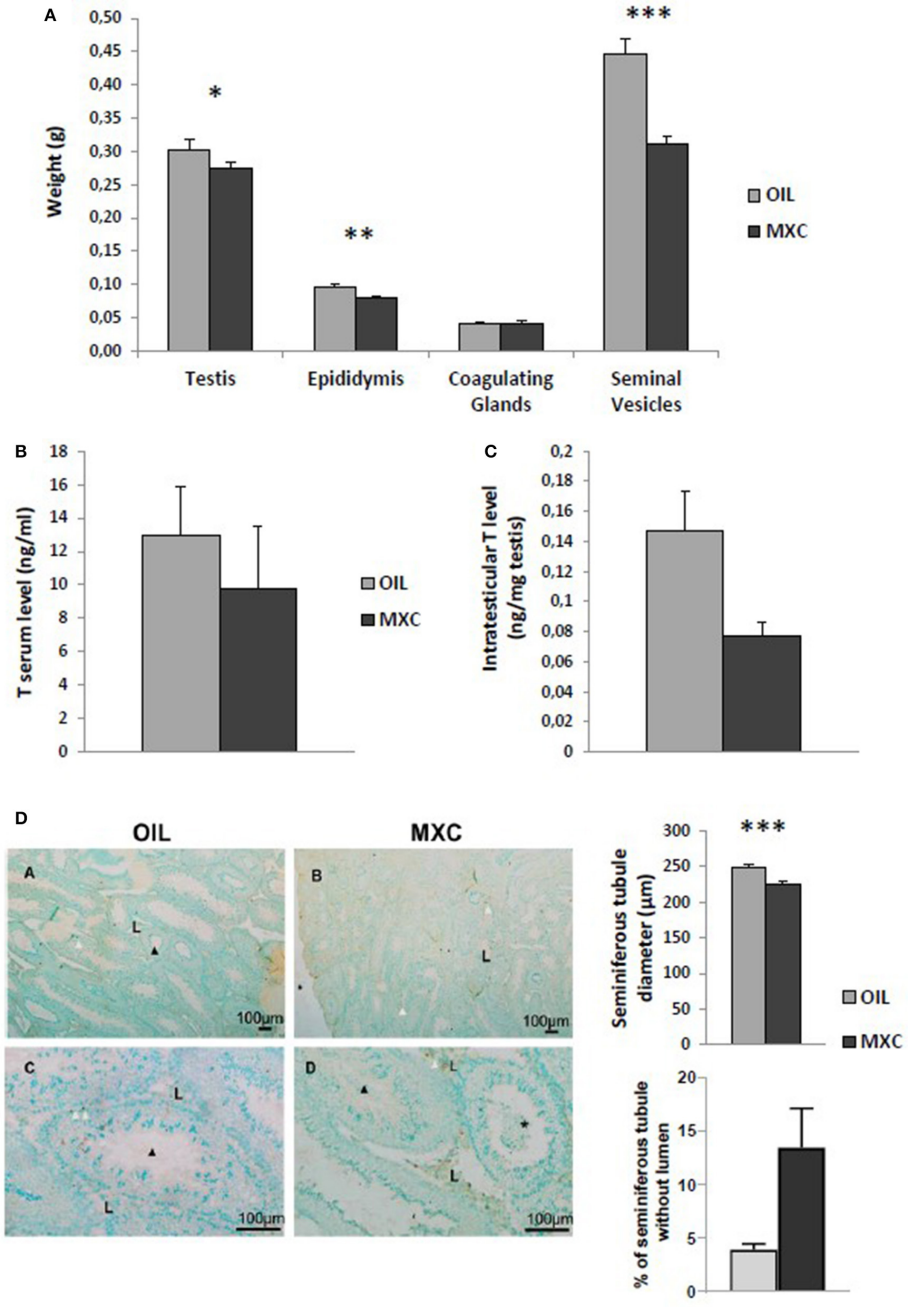
Effect of perinatal exposure to MXC on male reproductive parameters. MXC exposure induces a lower weight of some reproductive organs such as epidydimis or seminiferous vesicles **(A)**. When measuring testosterone (T) levels in both serum **(B)** and testes **(C)**, a significant difference was only observed only in testes. Finally, testicular morphology was altered in presence of MXC **(D)**. Administration of MXC lead to a decrease in the diameter of seminiferous tubules (MXC: 225 μm vs. control: 250 μm), an absence of lumen in some seminiferous tubule and presence of sloughed cells in the lumen of seminiferous tubules. Photographs: *, sloughed cells in the lumen; black arrow, spermatozoa, white arrow; TUNEL-positive cell; L, Leydig cells. For all graphs: *p < 0.05; **p < 0.01, ***p < 0.001 for OIL vs MXC exposure.

In males, *t*-test revealed no effects of MXC exposure in testis and coagulating glands weight between MXC exposed and control mice (*t*-value: −1.607, df: 22, *p* = 0.122 and *t*-value: 0.150, df: 22, *p* = 0.882, respectively). By contrast, perinatal MXC exposure significantly decreased the weight of the epididymis and seminiferous vesicles in exposed males compared to controls (*t*-value: −3.023, df: 22, *p* = 0.006 and *t*-value: −5.183, df: 22, *p* < 0.001, respectively) (Figure 3A). *T*-test on testosterone content in the testes revealed a significant difference between control and exposed males (*p* = 0.0194) (Figure 3B). However, this difference was attenuated in the periphery as *t*-test on testosterone serum level revealed no effects of MXC (*t*-value: 0.686, df: 21, *p* = 0.5) (Figure 3C).

Despite the lack of difference in the testes weight, testicular morphology was altered in presence of MXC. Administration of MXC led to a qualitative decrease in the diameter of seminiferous tubules, an absence of lumen in some seminiferous tubule (*p* = 0.0543, Figure 3D) and the presence of sloughed cells in the lumen of seminiferous tubules (Figure 3D). Finally, qualitative analysis of TUNEL labeling did not show any qualitative increase in cell death in the seminiferous tubules.

### Mate Preference Tests

**Figure 4:**
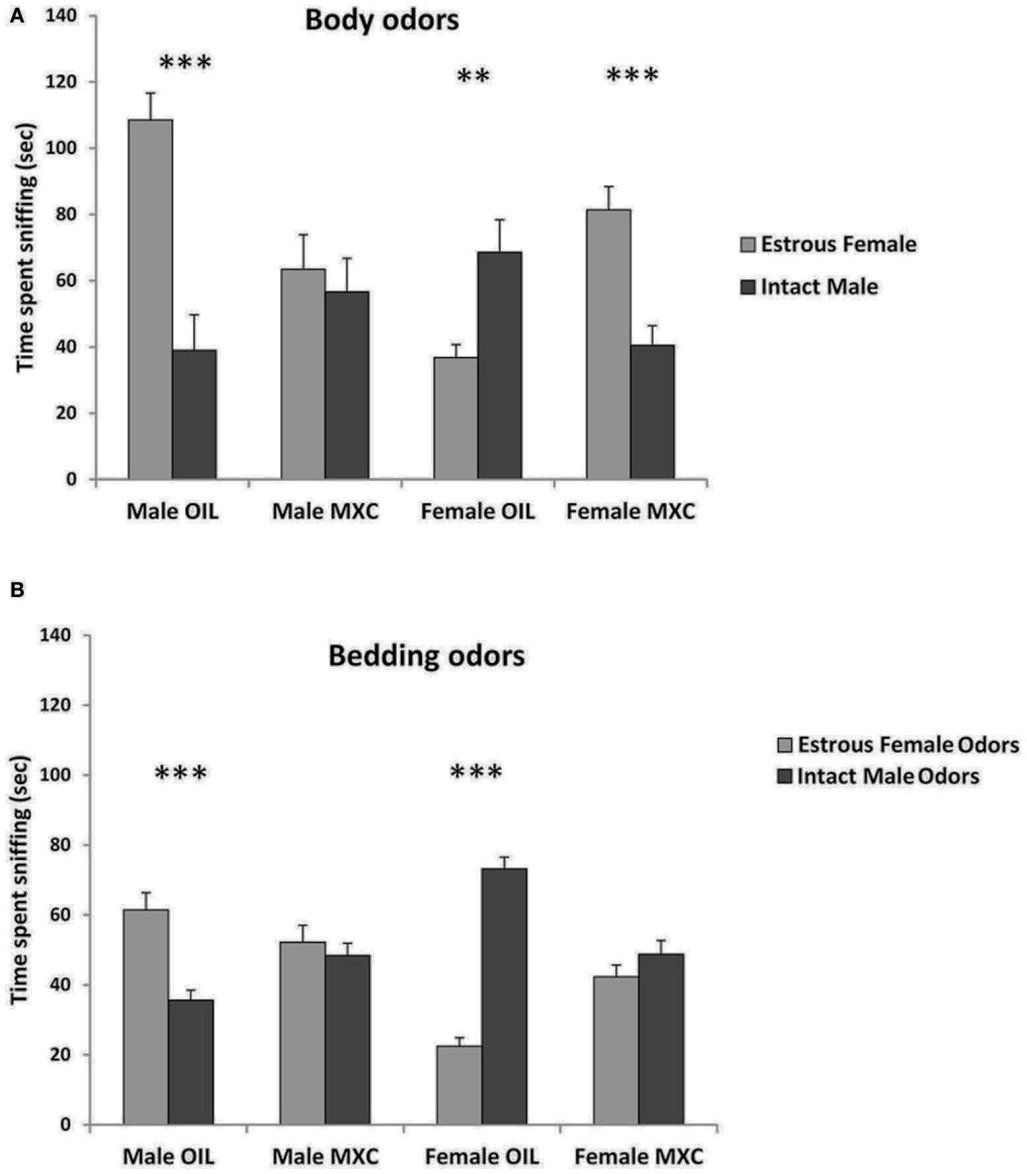
Tests of sexual preference. **(A)** Test of sexual preference using anesthetized conspecifics. Male mice treated with OIL show a typical preference for estrous female while MXC treated males do not show any preference. By contrast to OIL treated males, OIL treated females show a preference for male over estrous female and MXC exposed females show the opposite pattern of response (i.e. a preference for estrous female). **(B)** Test of olfactory sexual preference using soiled bedding by intact male vs. estrous female. When repeating the preference test using odors derived from soiled bedding rather than anesthetized conspecifics, the same pattern of response was observed with the exception of MXC treated female mice that now failed to show any preference for estrous female odor over intact male. For all graphs: **p < 0.01, ***p < 0.001 for OIL vs. MXC exposure.

A three-way repeated measures ANOVA, with sex and perinatal exposure as independent factor and the stimulus animal as repeated factor revealed a significant interaction stimulus animal^*^sex^*^treatment [*F*_(1,39)_ = 46.944, *p* < 0.001]. Indeed, when male mice were given a choice between body odors from intact male or estrous female (Figure 4A), control males spent a longer time sniffing the estrous female whereas males perinatally exposed to MXC spent a similar time sniffing the intact male and the estrous female (*t*-value = 5.181, df = 20, *p* < 0.001 for control males and *t*-value = 0.469, df = 20, *p* = 0.644 for males perinatally exposed to MXC).

When female mice were given a choice between body odors from intact male or estrous female (Figure 4A), control females spent a longer time sniffing the intact male, whereas females perinatally exposed to MXC investigated the estrous female longer than the intact males (*t*-value = −2.997, df = 20, *p* = 0.007 for control females and *t*-value = 4.464, df = 18, *p* < 0.001 for females perinatally exposed to MXC).

Regarding the bedding odors, a three-way repeated measures ANOVA, with sex and perinatal exposure as independent factor and the odor stimulus as repeated factor, revealed again a significant interaction odor stimulus^*^sex^*^treatment [*F*_(1,56)_ = 43.068, *p* < 0.001]. Indeed, when male mice were given a choice between soiled beddings from intact male or estrous female (Figure 4B), control males spent a longer time sniffing the estrous female whereas males perinatally exposed to MXC spent a similar time sniffing the intact male and the estrous female (*t*-value = −4.586, df = 28, *p* < 0.001 for control males and *t*-value = −0.638, df = 28, *p* = 0.529 for males perinatally exposed to MXC).

When female mice were given a choice between soiled bedding from intact male or estrous female (Figure 4B), control females spent a longer time sniffing the intact male, whereas females perinatally exposed to MXC spent the same time investigating the two soiled beddings estrous (*t*-value = 12.212, df = 28, *p* < 0.001 for control females and *t*-value = 1.255, df = 28, *p* = 0.220 for females perinatally exposed to MXC).

## Discussion

Our study confirms the effects observed in the literature about the effects of perinatal MXC exposure on reproductive function and sexual responses, especially in male mice. However, we show here that these effects can be observed at a much lower dose than previously expected, as the dose used in our study (20 μg/kg/day) is well below the reference doses (LOAEL: 50 mg/kg/day; NOAEL: 5 mg/kg/day) and the range of doses used in the literature (usually from 100 μg/kg/day to several mg/kg/day). To our knowledge, an effect at such low dose of MXC has been only evidenced so far for cognitive responses (26). As a whole, this result adds to the debate on the effects of very low doses of EDCs (46).

AGD is sexually differentiated with males having a larger AGD than females. Male AGD is well-determined by fetal concentrations of testosterone and measuring AGD is an established method for determining *in utero* exposure to testosterone (47). MXC exposed males had a lower AGD than control males, which also correlates with the lower T concentration, suggesting that the synthesis of T by the testis was also disrupted during development. This result in males is in accordance with Palanza et al. (21), which showed that injection of MXC, although at higher doses, during the last week of gestation, induced a reduction in AGD. By contrast, MXC induced an increase in female AGD, an observation already reported for a high dose of MXC in the study of Palanza et al. (21).

Then, we assessed pubertal development by measuring pubertal landmarks as indices of maturation of the hypothalamic-pituitary-gonadal axis. We showed a significant but temporary sex-specific effect of MXC exposure. Indeed, MXC treated females showed a small advance of vaginal opening between PD27-29 in comparison to control animals while MXC males showed a small delay in preputial separation around PD26-27. All these effects observed during development appear to be quite specific to reproductive function as no differences in body weight were observed between MXC and OIL treated animals from PD4 to PD120 and only a difference between the sexes was evident with males being ~20% heavier than females.

We then explored whether MXC exposure induced any differences in both female and male adult reproductive function, especially at the level of Kisspeptin neurons which are known to be a target of EDCs (48). MXC treated females exhibited a higher number of Kisspeptin cells in the AVPV preoptic region. By contrast, in males, Kisspeptin expression did not differ between MXC and OIL treated animals. However, this could be explained by the fact that Kisspeptin is sexually differentiated and males express only a very low number of cells, thus probably preventing to see any further decrease due to MXC exposure.

At the level of reproductive organs, no effects on female adult reproductive function were detected. Indeed, the stages of estrous cycle, the number of pups produced over a 3 months period when paired with a sexually active male, their sex-ratio and the weight of both uterus and ovaries did not show any statistical difference according to the treatment. This result seems surprising at first, given the literature reporting alteration of ovarian physiology following MXC exposure (49). It is possible that the low dose of MXC used here is without effect; however, studies consistently show that MXC inhibits ovarian steroidogenesis and follicle growth. This lack of effect does not exclude more subtle effects on other female reproductive parameters.

By contrast to females, we showed here a strong effect of MXC exposure on male reproductive function in accordance with numerous reports showing that exposure to MXC led to a decrease in testicular weight and activity (reduced androgen secretion), sperm count and motility (50, 51). Here, the weight of several androgen sensitive glands such as the epididymis and the seminiferous vesicles was also reduced following MXC exposure as well as the diameter of seminiferous tubes. This result suggest that the amount of spermatozoa produced could be potentially also reduced which would be in accordance with the reduced weight of the epididymis, a structure being involved in the storage of spermatozoa. In addition, the presence of cells in the lumen of seminiferous tubes is not normal and some seminiferous tubes did not present any lumen in MXC treated males. It should be remained that this process is androgen-dependent (52). Finally, a qualitative analysis of TUNEL labeling did not show any marked increase in cell death in males MXC. This seems to be in contrast with reports in the literature showing that another marker of apoptosis, caspase-3 activity, was significantly increased in a dose related manner following MXC exposure (53). However, this discrepancy could be due to differences in the markers used, with a higher sensitivity of caspase 3 activity being reported in comparison to TUNEL method. It is clear that other male parameters could have been performed to get more mechanistical insight. Such exploration could have explain, for example, the reduction in weight of the epididymis which is intriguing. This could be due either to a decrease in the sperm content of the cauda, or a decrease in tissue weight due to the reduction in stimulatory lumicrine androgens.

Finally, we assessed mate preference in males and females as this behavior is known to be finely tuned by the effect of perinatal testosterone (54, 55). We observed a strong effect of MXC treatment on mate preference tests performed on the basis of either whole body odors or bedding odors. Indeed, the male preference for estrus female odors is disrupted in MXC males and the preference for intact males expressed by females is also disrupted if not reversed. This result suggests that brain mechanisms controlling mate preference were also affected by MXC exposure. Interestingly, it has been recently shown that Kisspeptin/Kiss1R signaling in the anteroventral periventricular nucleus triggers olfactory-driven mate preference in female mice (56). Knowing that MXC exposure also alters Kisspeptin expression, it is possible that the disruption of mate preference in MXC exposed animals is linked with the changes observed in Kisspeptin expression. Further testing should be performed to assess whether MXC exposure are due to olfactory impairment or are acting at a more central stage than the olfactory system.

It should be reminded that the same MXC perinatal exposure led to an increase in anxiety-like behavior and in short-term spatial working memory in both sexes (26). These results were also correlated with an increased survival of adult generated cells in the adult hippocampus (26). Given these results, it is clear that the specificity of the behavioral deficits observed should be further confirmed and advocates for future studies exploring the effects of MXC on reproductive dependent behavior.

In conclusion, the present work shows that perinatal exposure to MXC affects pubertal development and adult mate preference in both sexes but also adult male reproductive function. These effects are observed at a very low dose in comparison to the vast majority of studies conducted so far, therefore highlighting the need for additional studies exploring the mechanistical effects of MXC exposure at these low doses.

## Data Availability Statement

The raw data supporting the conclusions of this article will be made available by the authors, without undue reservation.

## Ethics Statement

The animal study was reviewed and approved by Comité d'ethique en experimentation animale Centre Val-de-Loire.

## Author Contributions

MK and MM: study design, supervision, data acquisition, and manuscript redaction and analysis. PF, IF, DP, and SM-K: data contribution and manuscript redaction. EG and CC: data contribution. All authors validated the manuscript.

## Conflict of Interest

The authors declare that the research was conducted in the absence of any commercial or financial relationships that could be construed as a potential conflict of interest.
